# Buffalo Medical and Surgical Association

**Published:** 1889-12

**Authors:** W. H. Bergtold

**Affiliations:** Secretary


### PROCEEDINGS OF THE BUFFALO MEDICAL AND SURGICAL ASSOCIATION.

Reported by W. H. BERGTOLD, M. D., Secretary.

Stated meeting Tuesday, Nov. 12th, at 8.15 P. M., in the Library of the Polytechnic Institute, No. 9 West Mohawk street.

The President, Dr. A. A. HUBBELL, in the chair.

Dr. J. D. FLAGG was proposed for membership.

Dr. J. W. GROSVENOR then read a paper, entitled "The Therapeutic Value of Alcohol." [This paper will be published in full in a future number.—EDITOR.]

DISCUSSION.

Dr. J. H. PRYOR called attention to the fact that laterally there have appeared a number of articles on the use of alcohol in the various journals of this country. Many of these writers seem to hold extreme views, as did the present essayist of the evening. To say that alcohol is used by the profession to-day in a too free and lavish manner, is, in Dr. Pryor's opinion, not true, and does to the members of the profession generally an injustice ; though, at the same time, it is not to be denied that the use of alcohol may be much abused, and that we, to-day, occasionally find a physician who thus errs. While there can be no doubt that in times past too much alcohol has been used as a medicine, it is not to be denied that its employment is being gradually dropped, and that its true position as a therapeutic agent is becoming better appreciated and known. The present writer makes a mistake, as have many others, in applying deductions drawn from experiments on healthy people and animals, to persons who are ill. This is entirely faulty and, instead, we ought only to base our arguments on facts observed from the use of alcohol clinically. That it is a poison no one attempts to deny, but, however, it is only so when used in quantities greater than its therapeutic dose, or in conditions not of disease ; it is no more a poison than are dozens of other drugs when used in a parallel manner. If alcohol be a food at all, it is a sustaining one, and one not of the reconstructive variety. Alcohol has no peculiar effect on any particular disease, but we use it for a general effect in many diseases. It acts to prevent tissue waste, either directly or through the nerve centers. In typhoid fever it acts markedly as a stimulant, not as a depressant. It is a narcotic when given to a healthy organism, when given where there is no indication, or when administered in too large a dose. Its antipyretic effect is exhibited almost exclusively when given in a condition of health. Those effects upon the blood which have been ascribed to it, ought not to occupy our attention even momentarily, for we never give it in quantity sufficient to approach a percentage in which blood changes are seen. Statistics, while useful in certain ways, are not always to be accepted as final, and this seems particularly true concerning the use of alcohol in typhoid fever.

In typhoid fever, alcohol is only to be used when certain conditions arise which are rather difficult to formulate: in this disease it certainly lowers the pulse, clears the tongue, lowers temperature, and tides over grave crises. For a best exhibition of these effects, it ought to be used in small doses, frequently repeated. In delirium tremens its use ought, in the majority of cases, to be discontinued at once. There is, however, a class of cases where this practice would be very injurious and injudicious; these are the cases of chronic alcoholics, who have been accustomed to its use for years. In shock it is of marked value, to be given in small doses, for, if otherwise, the stomach often refuses to accept it all.

Dr. Strong was much pleased with the paper, and the opening of the discussion.

He believes that it is wrong to condemn alcohol because we call it a poison, and that it is a mistake to argue for its expulsion from effects produced on the healthy person.

Its value in some diseases is, in his mind, established beyond controversy. He was disappointed that the essayist had not mentioned its use in tuberculosis, and in other conditions of excessive secretions, as in the bronchitis of the aged. It is possible to set up the alcohol habit by its free use, and we should, therefore, be cautious to whom we give it.

Dr. Bartlett said he had never brought on the habit by its use. He would feel at a loss if its use were prohibited. It is of particular usefulness in snake bites, and it is also given with pleasant results in persons of melancholic habit. Its position relative to typhoid fever is uncertain.

Dr. Burghardt expressed the opinion that we should always be cautious in its use. He had seen a case where it undoubtedly produced an attack of apoplexy.

Dr. Brecht was formerly accustomed to use alcohol considerably in cholera infantum, diphtheria, and typhoid fever, but latterly he has not employed it nearly so much, preferring other stimulants, which, in his experience, seem to act just as well. He wished to raise his voice against the giving of beer, etc., to children, a practice particularly common with the Germans.

Dr. Van Peyma.—While he was much pleased with the paper, yet it struck him as having been written with too much of a preconceived notion, instead of a desire to make an impartial review. He, also, thinks that alcohol should be used in small doses, frequently repeated. To his mind, it is very difficult to clearly define the line between stimulants and narcotics. He has found that beer is often of much use as a stimulant to the appetite. Here, as with many of the wines, the resulting effect is not due to the contained alcohol alone, but also to the various bitters, aromatics, etc. With Dr. Pryor, he agrees that in old persons suffering with delirium tremens, the use of alcohol ought not to be suddenly discontinued.

Dr. Hubbell was much interested in the paper, and in the main concurred with its points. He did not think that absolute reliance could be placed upon statistics, and particularly those relating to typhoid fever, for here there are so many diverse factors to be considered.

Dr. Grosvenor, in closing the discussion, said that notwithstanding the results of the evening's debate, he still believed that too much alcohol was prescribed by the profession. If we admit that it is a poison, then we are in duty bound to exercise as much care in its use as with any other poison. While in some diseases the effects of a drug are different from when used in a normal condition, yet some drugs exhibit very similar effects when used in a normal as in a pathological condition.

If an observer compiles a set of statistics, and has confidence in them himself, it seems but fair for us to at least have some trust in the statistics of others; while statistics may not be reliable, yet, for that matter, very few things in this life are. In the delirium tremens of old people he has had but little experience, and he can imagine that in these cases the sudden withdrawal of alcohol might be deleterious, yet this is not at all so with young patients.

The next meeting will be held Tuesday evening, December 3, 1889. Subject: Chorea,—Its Etiology and Relations to other Diseases, by Dr. M. B. Folwell; Its Pathology, by Dr. S. Y. Howell; Symptoms, Diagnosis, Prognosis, and Treatment, by Dr. H. D. Ingraham. To be further expressly discussed by Drs. Crego, Hopkins, Putnam and Krauss.

---

He Had Been There.—George: "What a fine building across the street." Charles: Yes; but the owner built it out of the blood, aches, and groans of his fellow-men." "Ah! A rumseller, I suppose?" "No. A dentist."—*Toronto Grip.*

